# Supplementary material for: Hierarchical effects facilitate spreading processes on synthetic and empirical multilayer networks
Source: PLoS One. 2021 Jun 9;16(6):e0252266. doi: 10.1371/journal.pone.0252266 (PMC8189515; doi:10.1371/journal.pone.0252266)
Supplement: S1 Appendix — Analysis of the hierarchical spreading model on a Polish Manufacturing network as a secondary empirical source to supplement the presented SNL data. (ZIP) [file pone.0252266.s001.zip › S1_Appendix.pdf]

S1 Appendix. Background plots, elaborations, and Polish manufacturing data.

Background plots and elaborations

State dynamics and parameterization

The state transitions of the Dosage Model studied here closely follow the standard SIR dynamics common to this type of study, with key differences brought about by the inclusion of individual memory in the model allowing for metastable states and long persistence times of the I compartment. As noted in [1], incorporation of memory increases the complexity of the model dynamics, which leads to an intractable analytical solution for the class of dynamics in this model. However, utilizing numerical simulation similar to that conducted in the original paper by Dodds and Watts, we have created average timeseries plots, shown in Fig S1.1. This plot shows the dynamics for the various cases of study in our system, all with seed size $0.015N$ as in the analysis in the paper. The curves are unsurprisingly qualitatively similar to the standard SIR model but with the longer tail characteristic of the dosage variant used here. We include this plot here to increase clarity on the general time dynamics of the simulations while the main paper provides a more in depth study of the characteristic features of these simulations. simulations. Namely, the main paper describes in greater detail the maximum infected population (Fig. 3 and Fig. 5 of main paper) and the persistence of the tail (timeseries plots in Fig. 6 of main paper).

Fig S1.1. State dynamics over time for the SNL networks. The timeries of the state dynamics for each of the test cases for the SNL networks. Seeded to $0.015N$ and averaged over 1000 simulations allowed to run for 1000 system timesteps.

It should be noted that the innovation can reach full, absorbing consensus only in the SI analogous cases where the removal rate is zero (shown in Fig 4 of the main paper). In the long time limit, for any model with an R state, the only possible end state of the system is the eradication of the infection as the lack of infection yields the only static state of the system. However, as shown in the persistent tails of the infected popluations in each case of Fig S1.1, with the individual memory introduced by the dosage model the system can reach metastable states that lead to surprisingly long tails as shown in Fig. 6 of the main paper.

The persistence of this metastable state is controlled via the system parameters used in each simulation [1]. For the case investigated here with a nonzero removal rate, an absorbing R state ($\rho = 0$ where ρ is the probability of moving from state R to state S) and a memory window of $T > 1$ the system stays within a single class of model as described in (Dodds 2005) for all values of $T > 1$ and $1 > r > 0$. In that paper, they investigate the critical points and phase transitions that arise from different parameter values, including this class of model, and show that with a removal rate of $r = 0.2$, as used in our model, varying the time window simply has the effect of increasing the tail of the simulation and lengthening the time spent in the metastable state. Futher, Dodds and Watts show that the effect of the timing window on the persistence time of the infection is an exponential relationship across values of T, where $t_{max} \propto \exp{(\alpha T)}$ (Eq 29 in their paper), indicating that increasing this timing window simply extends the tail of the simulation.

Synthetic hierarchical networks

The synthetic networks used here utilize a two-layer hierarchy consisting of a staff layer and a management layer. First the staff layer is created (here using a Random Geometric Graph) that is then divided into a set number of communities c via the Fluid Communities algorithm [2]. The creation of the manager layer is done via the creation of new nodes that are spawned as a secondary network, then interconnected to the staff network with interlayer edges only connecting managers to a single downstream community each. The number of nodes in the management layer is exactly equal to the number of partitions created in the staff layer, $m = c = 0.06N$. This is exactly analogous to the interlayer connections used in the empirical datasets, and the intralayer connections between the managers are created independently based on the schemes described (including no connections, full connections, ER, BA, and RGG graphs). For the sort of organizational structure studied here, the size of the downstream group and the degree of the node in the upstream layer are not strongly correlated (as shown in Fig S1.2) and thus, we do not consider any importance measures for managers when assigning communities for manager attachments (see S2 Sample Code for an example of this process). For other organizations and contexts, it may be prudent to utilize node importance or centrality measures when assigning downstream communities.

Fig S1.2. The downstream vs intralayer degrees of nodes in the first management layer of the SNL Line-Org network. The correlation between downstream and intralayer degree for the fist management layer of the SNL Line-Org network, showing the correlation of intralayer importance versus size of managed group for the first management layer in the empirical network).

The staff level communities created via the Fluid Communities algorithm are consistent in size and number of communities. In each simulation case there were exactly $0.06N$ partitions (for the case of system size $N = 1000$ there were 60 communities), and the communities generated from this process have an average size of 16.667 nodes with a standard deviation of 5.642 and are distributed as a skewed normal distribution with skewing parameter $\alpha = 2.600$, location $\xi = 10.417$ and scale $\omega = 8.420$. This fit is exemplified in Fig S1.3, where we show the occurrences of different community sizes within the generation of 10000 networks, each of size $N = 1000$ and divided into 60 community partitions (as in the main paper) for a total sample of 600,000 communities compared to the distribution.

Fig S1.3. Distribution of communities in synthetic hierarchical network. Here we show the fit of the communities produced by the Fluid Communities algorithm with a skeweed normal distribution, exemplifying the expected community sizes that are used in the synthetic hierarchical network scheme.

Polish manufacturing network.

While the laboratory network used in the main article represents a novel empirical network to test the proposed model on, it also represents only a single representation and size of such a corporate hierarchy network. In the interest of results reproduction, we repeat the main experiments from the main text of the paper on a publically available Polish manufacturing dataset [3, 4]. This dataset is a 167 node network of employees that work at a manufacturing plant in Poland. It contains the direct reporting structure as well as the quantity of emails sent between employees over a nine month span in 2010. For the purposes of comparison to the main paper, we utilize this

data to build two analogous networks to those built from the SNL dataset. The dataset is similar in many ways to the SNL dataset, but much smaller, allowing for a useful comparison points to ensure results hold in small organizations. There are five total layers, and when accounting for removing disconnected components there are a total of 154 nodes: 120 staff, 31 Level 1 Managers, and one node for Levels $3-5$ management.

First, we utilize the reporting structure to build a Manufacturing Line-Org network, connecting management with direct subordinates as well as implementing complete graphs between the subordinates underneath a single manager. For the purposes of network levels, managers are assigned a level equal to the depth of the management chain below them. For example, a staff node with no subordinates is considered to be the bottom layer of the network, while a node with a single layer of subordinates beneath them is considered to be a Level 1 Manager in the second layer of the network.

The second network, built in analogue to the Project network in the SNL networks, utilizes the email data to build an interaction social network, the Manufacturing Email network. To this end, we temporally flatten out timescale in order to obtain a static network where we deal solely with the total volume of messages send between nodes. Then, we impose an edge threshold of 34 emails such that only nodes that contain at least 34 emails between are connected in the network. The threshold 34 is chosen, as in the body of the main paper, to enforce an equal average degree for the Polish Email network and the Polish Line-Org network.

Table S1.1. Network statistics of empirical manufacturing networks by layer.

	**Manufacturing Line-Org**			**Manufacturing Email**		
	$\langle C_{centrality} \rangle$	$\langle k \rangle$	$\langle C_{clustering} \rangle$	$\langle C_{centrality} \rangle$	$\langle k \rangle$	$\langle C_{clustering} \rangle$
Layer 1	0.26	6.61	0.94	0.34	6.30	0.36
Layer 2	0.33	12.10	0.55	0.39	13.97	0.26
Layer 3	0.39	12.00	0.52	0.33	5.00	0.30
Layer 4	0.45	19.00	0.49	0.46	21.00	0.25
Layer 5	0.37	13.00	1.0	0.44	16.00	0.30
Total	0.28	7.87	0.86	0.35	8.00	0.34

Shows various centrality and clustering statistics for each empirical network to inform on the structure, where $\langle C_{centrality} \rangle$ is the average closeness centrality, $\langle k \rangle$ is the average degree, and $\langle C_{clustering} \rangle$ is the average clustering coefficient for the nodes in each layer.

As shown in Table S1.1, these networks show a qualitatively similar structure to the SNL networks, although with lower degree overall and a more convoluted management scheme and less severe management skew in the Email network than the comparable SNL Project network. Considering the size of the networks, the degree to which the edges skew towards the managers is remarkably similar to the SNL dataset. The centrality and clustering are slightly more flat than is seen in the SNL dataset, but overall the two systems are very comparable. The biggest structural differences come from the much smaller size, leaving the network much more dense and shortening the paths from node to node.

Fig S1.4. Max adoption on Manufacturing networks with SIR dynamics. Shows the average maximum population of nodes in state I for each combination of network, seed size, and seed level, averaged over 1000 runs. Includes a 'flat network' consideration, where management advantages are removed. Larger seed sizes for management levels 2 and 3, since there is only a single node in those categories.
A: Average max reach in the Manufacturing Line Org network structure. B: Average max reach in the Manufacturing Email network structure.

Utilizing the same dynamics as outlined in the body of the main paper, we tested the average maximum infected population at different seed levels, shown in Fig S1.4. The advantages of the less centralized Email network still provide a large increase in maximum adoption over the line-org, but with a much denser and less tree-like management structure in this dataset, the Line-Org scheme outperforms its SNL analogue. In all cases, however, incorporating hierarchical dynamics within the model significantly outperforms the flattened network consideration, even when the seeding is only done on the base layer. In these networks, the smaller size of the network means that a minimal seed is not quite the hindrance that it was in the much larger SNL networks, however comparing the case where the seed is at 1.5% and 3% shows that the advantage of larger seeding saturates quickly and shows lower returns for increased seed size.

Fig S1.5. Stability of I population in long-time scenarios for the manufacturing networks. All seeds are size $0.15N$ and all data points are averaged over 1000 runs.
A: The surviving percentage of the maximum infected population over time. B: The raw percentage of nodes in state I over time.

Finally, we repeat the analysis for the long-time tails of the infection, showing an interesting reversal in the trends seen in the main paper. In this case, the Manufacturing Line-Org network, which is more centralized than its SNL counterpart, has a fatter tail in the long time than the Manufacturing Email network. Similar to in the SNL networks, when looking at the percentage of the infected community, the staff seeding slightly outperforms the management seeding, echoing the sentiment of using management as first adopters being slightly unoptimal. It should be noted, however, that neither scheme is able to level off at a relatively stable community like the SNL Project network, and with the much smaller network size these surviving communities are of negligible size and not necessarily representative of slow-dying pockets of nodes. Indeed, rather than an over-performing Manufacturing Line-Org network it appears that this change is driven by a poorly performing Email network, likely driven by the lower skew towards the managers making it a less centralized network than the SNL Project network. Additionally, while there would expected to be high overlap, it is worth noting that the co-working scheme that the Project network draws from is *not* the same as a communication network like the Polish Email network. While they have many similar features, anomalies such as the uncharacteristic low degree of the Level 2 manager relative to the other managers in the Email network may be indicative of its less optimal structure derived from communication patterns that do not normally align with co-working tendencies.

References

1. Dodds PSS, Watts DJJ. A generalized model of social and biological contagion. Journal of theoretical biology. 2005;232(4):587–604. doi:10.1016/j.jtbi.2004.09.006.

2. Parés F, Gasulla DG, Vilalta A, Moreno J, Ayguadé E, Labarta J, et al. Fluid Communities: A Competitive, Scalable and Diverse Community Detection Algorithm. Springer, Cham; 2018. p. 229–240. Available from: `http://link.springer.com/10.1007/978-3-319-72150-7{_}19`.

3. Michalski R, Kajdanowicz T, Bródka P, Kazienko P. Seed Selection for Spread of Influence in Social Networks: Temporal vs. Static Approach. New Generation Computing. 2014;32(3-4):213–235.

4. Bender-deMoll S, Morris M, Wang L, van de Bunt G, Bossaert G, Meidert N, et al.. networkDynamicData: Dynamic (Longitudinal) Network Datasets; 2016. Available from: https://cran.r-project.org/web/packages/networkDynamicData/.
