# Supplementary material for: Hierarchical effects facilitate spreading processes on synthetic and empirical multilayer networks
Source: PLoS One. 2021 Jun 9;16(6):e0252266. doi: 10.1371/journal.pone.0252266 (PMC8189515; doi:10.1371/journal.pone.0252266)
Supplement: S1 Sample code — Sample code for generating the hierarchical synthetic networks used in this paper. The code is written in Python and uses the NetworkX package for the creation of established network structures and community detection implementation [48]. (ZIP) [file pone.0252266.s002.zip › S1_Sample code.pdf]

S2 Sample Code. Synthetic network creation python script.

```
import networkx as nx

#hierarcichal synthetic network build function
#builds an RGG base (staff) layer
#then builds in manager nodes for each community
#n is size of the base staff layer
#m is the number of partitions and manager nodes to add
#k is the average degree desired on each layer
def synthetic_network_build(n,m,k):
   #initialize base layer network
   done=False
   while done==False:
      network = nx.random_geometric_graph(n, radius=(k/(n*3.14159))**(1/2))
      if nx.is_connected(network):
         done=True
   for nodes in network:
      network.nodes()[nodes]['level']=1

   #divide network into communities
   parts = list(community.asyn_fluidc(network,int(m)))

   #create new network for manager nodes
   #for random geometric graph
   if man_type == 'rgg':
      managers = nx.random_geometric_graph(len(parts),
          radius=(k/(m*3.14159))**(1/2))
   #for fully connected graph
   elif man_type == 'fc':
      managers = nx.complete_graph(len(parts))
   #for Erdos-Renyi graph
   elif man_type == 'er':
      managers = nx.erdos_renyi_graph(len(parts), k/m)
   #for Barabasi Albert graph
   elif man_type == 'ba':
      managers=nx.barabasi_albert_graph(len(parts),k/2)
   #for no connections between managers
   elif man_type == 'nc':
      managers = nx.empty_graph(len(parts))

   for nodes in managers:
      managers.nodes()[nodes]['level']=2

   #combine networks
   network = nx.disjoint_union(network,managers)

   #find manager
   index=0
   for nodes in network:
      if(network.nodes()[nodes]['level'] == 2):
         index = nodes
         break

   #add one manager per group
```

```
for group in parts:
    current = list(group)
    #for each node in community add edge to the new manager
    for nodes in current:
        network.add_edge(nodes, index)
    index += 1

return network
```
