# Supplementary figures and images for: Hierarchical effects facilitate spreading processes on synthetic and empirical multilayer networks
Source: PLoS One. 2021 Jun 9;16(6):e0252266. doi: 10.1371/journal.pone.0252266 (PMC8189515; doi:10.1371/journal.pone.0252266)

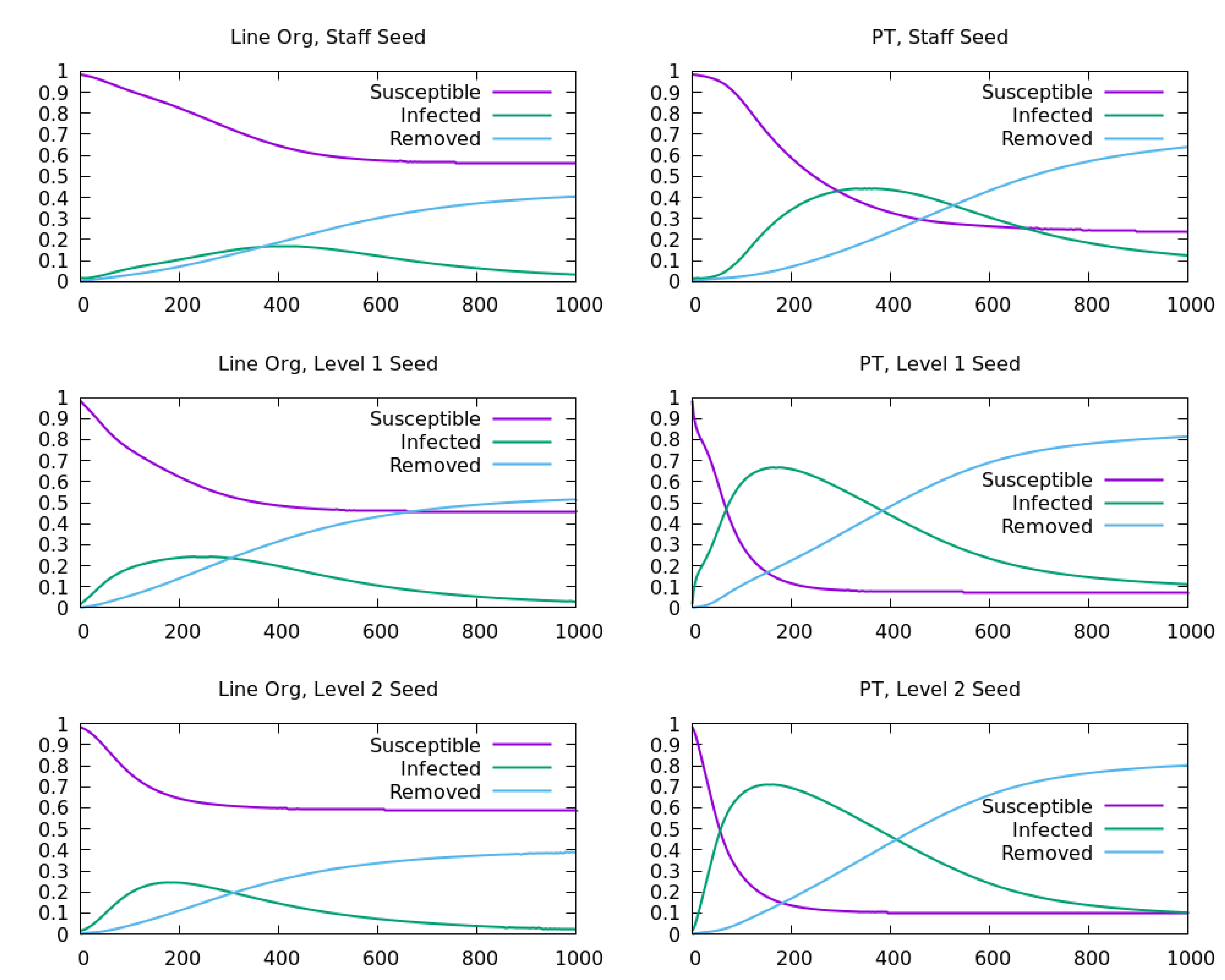

Supplement: S1 Fig — (TIF) [file pone.0252266.s003.tif]

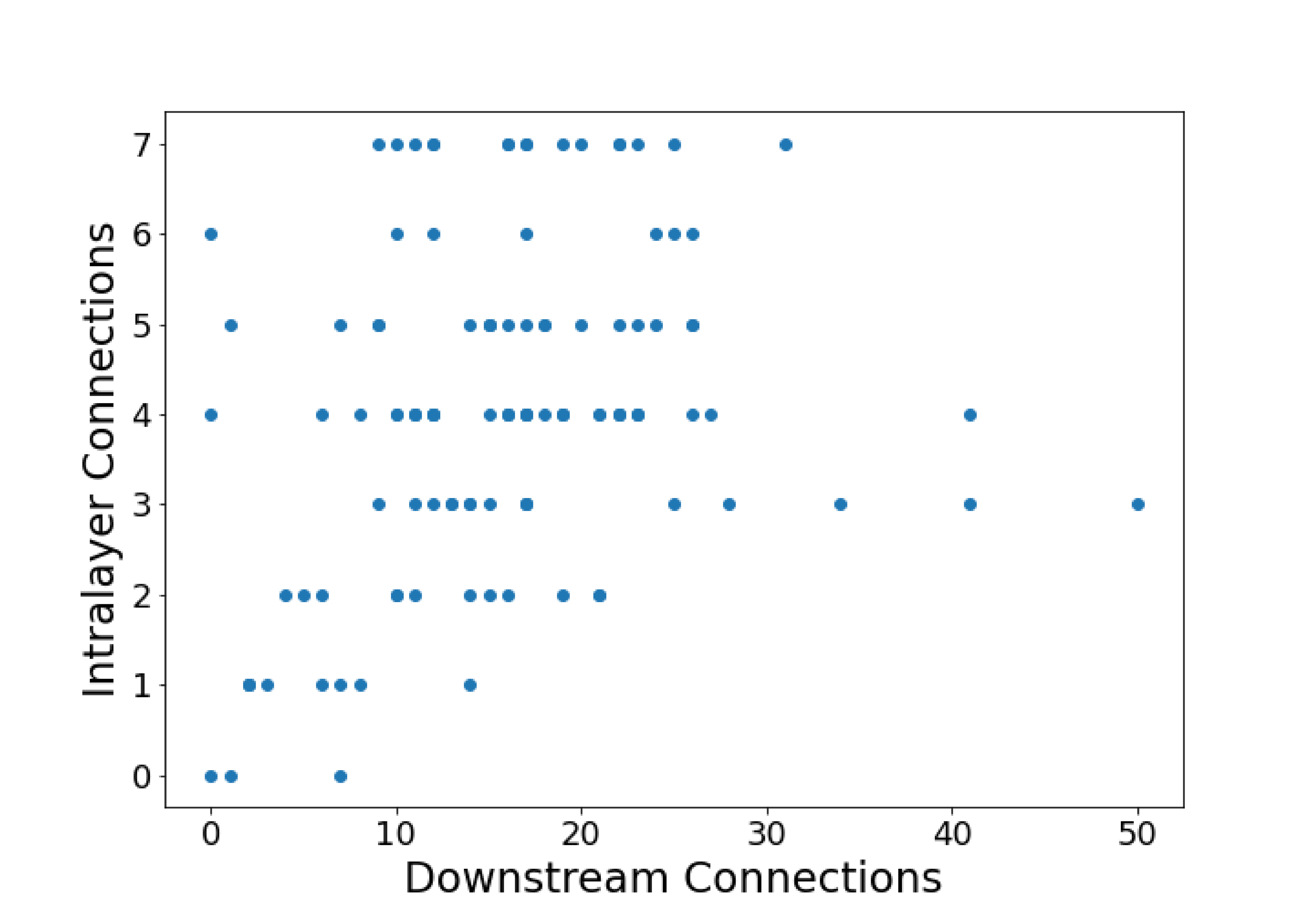

Supplement: S2 Fig — (TIF) [file pone.0252266.s004.tif]

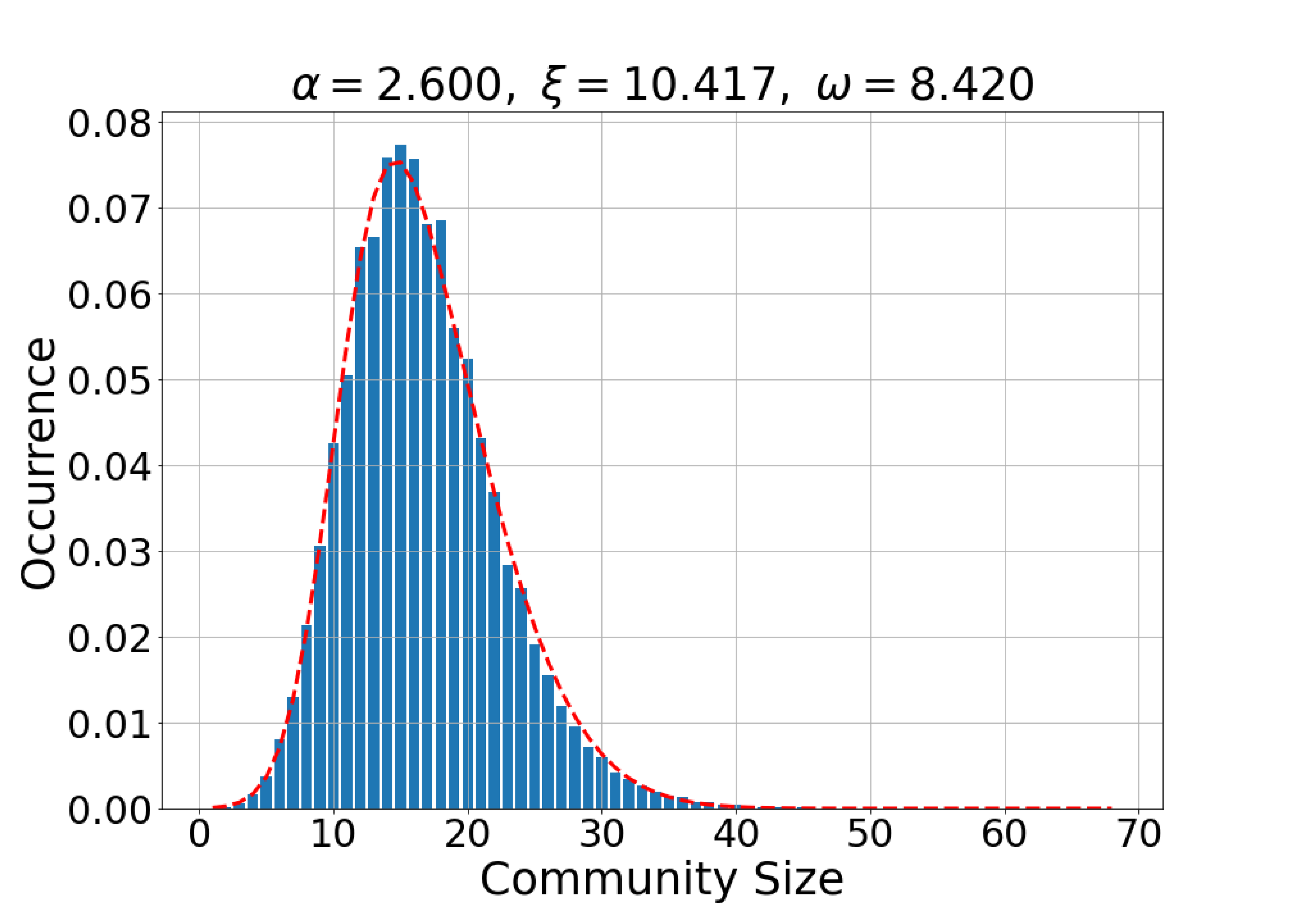

Supplement: S3 Fig — (TIF) [file pone.0252266.s005.tif]

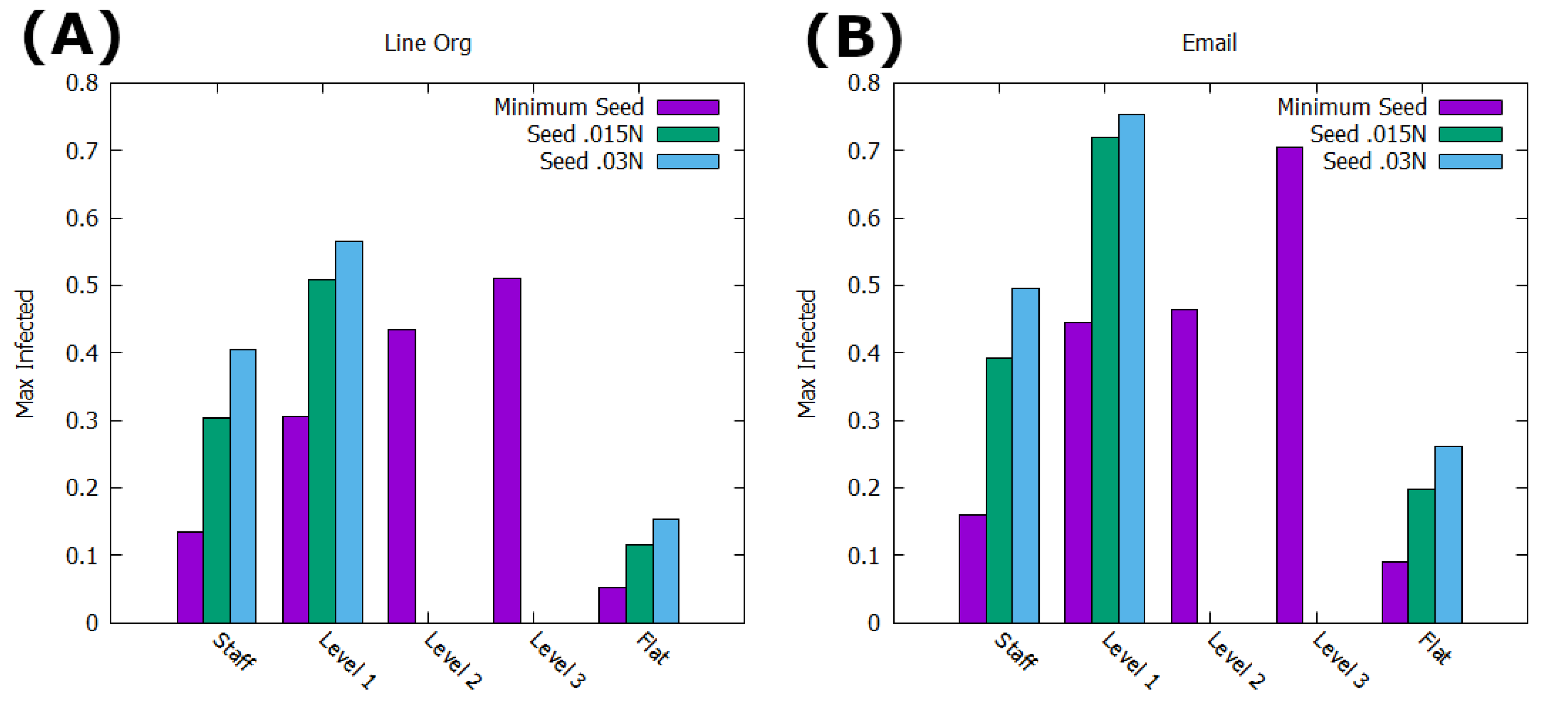

Supplement: S4 Fig — (TIF) [file pone.0252266.s006.tif]

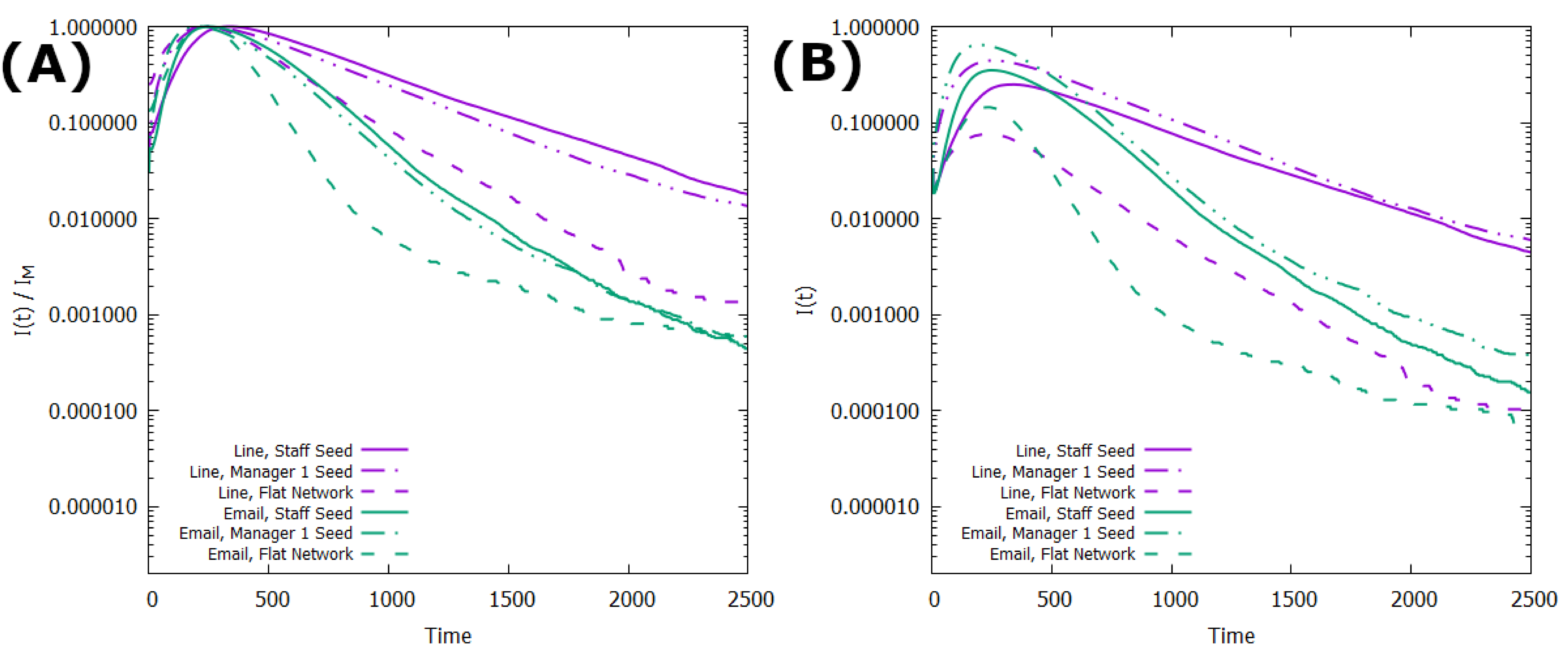

Supplement: S5 Fig — (TIF) [file pone.0252266.s007.tif]
