# Supplementary material for: Hierarchical effects facilitate spreading processes on synthetic and empirical multilayer networks
Source: PLoS One. 2021 Jun 9;16(6):e0252266. doi: 10.1371/journal.pone.0252266 (PMC8189515; doi:10.1371/journal.pone.0252266)
Supplement: Attachment — Submitted filename: Response to Reviewer.pdf [file pone.0252266.s016.pdf]

Casey Doyle
Sandia National Laboratories
P.O. Box 5800
Albuquerque, NM 87185-1137

Phone: (505)-845-0055
email: cldoyle@sandia.gov

May 10, 2021

Joerg Hever, PhD
PLOS ONE
1160 Battery Street
San Francisco, CA 94111

Dear Dr. Heber:

We are glad the reviewer is satisfied with the content of the paper, and believe with your help the paper is much stronger now than it was at initial submission. We have added the new appendix requested to ensure that the clarifications made are available to readers of the published article as well. We include here a response to the final reviewer request, as well as all of the prior exchanges preserved as ”Round One” and ”Round Two” responses. In the marked paper, edits from the original manuscript are codes as violet for first round edits, blue for second round edits, and red for new material from the third round edits.

Sincerely,
Casey Doyle, PhD
Sandia National Laboratories
R&D, Systems & Engineering
Systems Research and Analysis

Reviewer Round Three Comments

1. **The paper has been further improved and in my opinion is ready for publication; however, I would like the authors to include the materials from their round 2 replay (those not included in the main paper) as a supplementary material (they can extend S1 or add a new supplement), since I think some of them might help the reader to faster understand some elements, for example one look at fig 1 and 2 from the replay explains a lot.**

   We appreciate the perspective of the reviewer and have added the content of the Round Two reviewer response to the paper at the beginning of S1 Appendix. We have chosen to combine the appendices for ease of reading and organization of figures and tables. We have included the full discussion of the state change dynamics, tail behavior and parameter sensitivity, as well as elaborations on the synthetic network creation and community size distribution. These elaborations are referenced in the main paper in lines $110 - 112$ for the state change and tail behavior mechanics as well as lines 183 and 184 for the synthetic network creation discussion.

Reviewer Round Two Comments

1. **The model description is still not detailed enough with regards to motivation and reasons behind using SIR to spread innovations e.g. Can the innovation be ever accepted or at the end, it will always be rejected by the population? How this relates to real-world cases?**

   The innovation can reach full, absorbing consensus only in the SI analogous cases where the removal rate is zero (shown in Fig 4). In the long time limit, for any model with an R state, the only possible end state of the system is the eradication of the infection as the lack of infection yields the only static state of the system. In other terms if there is a live infection spreading, there is always a chance that it will die out, but once it has fully died out there is no chance of it reemerging without outside seeding. Over long enough time this forces a state composed entirely of S and R individuals. However, with the individual memory introduced by the dosage model, the system can reach metastable states that lead to surprisingly long tails on the lifespan of the infection which is a key consideration we investigate here as shown in Fig. 6. More information on this dynamic can be seen in (Dodds 2005). In real world cases this relates to a disease or innovation that is not self-sustaining, or externally forced. For our purposes this means an efficiency neutral or worse innovation where individuals have no drive to continue usage other than social pressures. Since our study is on the effects of these social pressures, using a neutral framework allows us to examine only those effects on the system. Conversely, an innovation that improves the efficiency of its users would need be implemented in such a model by external forcing, spontaneous adoption (unrelated to agent interactions), or refusal to abandon the innovation ($r = 0$) and the subsequent analysis would have to focus on the size those effects have on the system. Clarification on this justification has been added in lines 142-143, and more information on the long-time behavior is included in the paper in lines 98-104.

   Maybe Fig 1 could extend (by adding 1b) by adding a theoretical SIR states dynamics – something like this
   `https://en.wikipedia.org/wiki/Compartmental_models_in_epidemiology#/media/File:Graph_SIR_model_without_vital_dynamics.svg`
   or this
   `https://en.wikipedia.org/wiki/Compartmental_models_in_epidemiology#/media/File:SIR_trajectory.png`
   and discuss this, also extend Fig 6 could be extended in the same way, by adding the state dynamics from simulations.

   As noted in (Dodds 2005), such an analytical solution to this model is intractable due to the complexity of the model for this class of dynamics. However, utilizing numerical simulation similar to that conducted in their paper, we can create average timeseries plots as requested by the reviewer. Here we show such timeseries dynamics for the various cases of study in our system, all with seed size $0.015N$ as in the analysis in the paper. The curves are unsurprisingly qualitatively similar to the standard SIR model but with the longer tail noted as the key characteristic of the dosage model used here. Due to the expected shape and well known dynamics of the standard SIR model, we do not include these plots in the main paper in favor of including analysis of their characteristic features; the maximum infected population which is described in Figs 3 and 5 and the persistence of the tail which is highlighted in the timeseries plots in Fig. 6. These clarifications, both on the long time behavior and the shape of the state curves, are noted in the paper in lines 98-104.

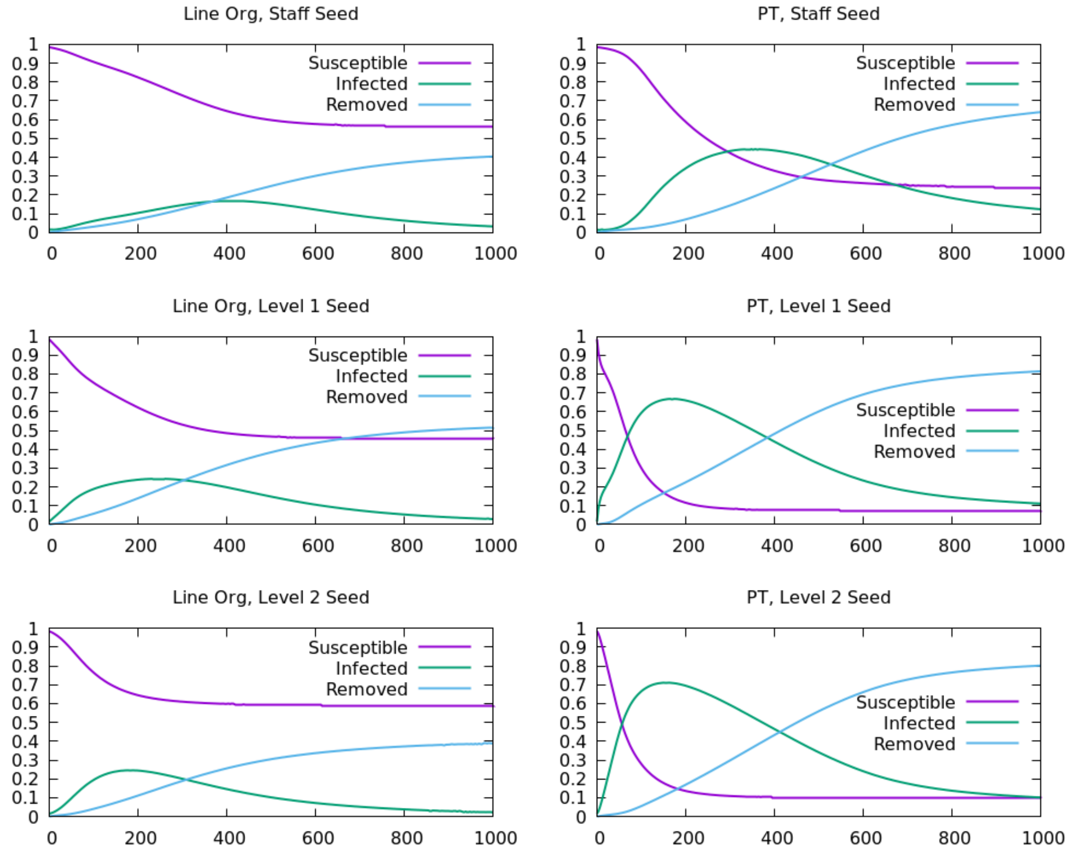

According to line 132-133, "there is no state transition directly from I to R under the assumption of good faith on the part of the agents". Should not it be from S to R since in line 91-93 we have "Conversely, if they are in state I and $Dti < di$, they have a probability $r = 1 = T$, or $r = 0.2$, of entering the removed state (R), signifying that they have abandoned the new innovation due to perceived lack of support."?

Yes, that was a critical typo in the manuscript and an important point to correct. This correction has been made and marked.

Citation:

Dodds, P. S., & Watts, D. J. (2005). A generalized model of social and biological contagion. Journal of Theoretical Biology, 232(4), 587–604. https://doi.org/10.1016/J.JTBI.2004.09.006

2. **Since the $c = .06N$, please provide the number of partitions in each simulation case. What is the distribution of partitions sizes? In line 150, please change N_{staff} to N or introduce what N_staff means.**

For each simulation case there were exactly $0.06N$ partitions (for the case of system size $N = 1000$ there were 60 communities). N_{staff} is the number of nodes in the base level of the system, not including the added management nodes. The communities generated from this process have an average size of 16.667 nodes with a standard deviation of 5.642 and are distributed as a skewed normal distribution with skewing parameter $\alpha = 2.6$, location $\xi = 10.417$ and scale $\omega = 8.420$. This can be seen here in the occurrences of different community sizes within the generation of 10000 networks, each of size $N = 1000$ and divided into 60 community partitions (as in the main paper) for a total sample of 600,000 communities compared to the distribution

fit. This information has been added to the paper in lines 161-170.

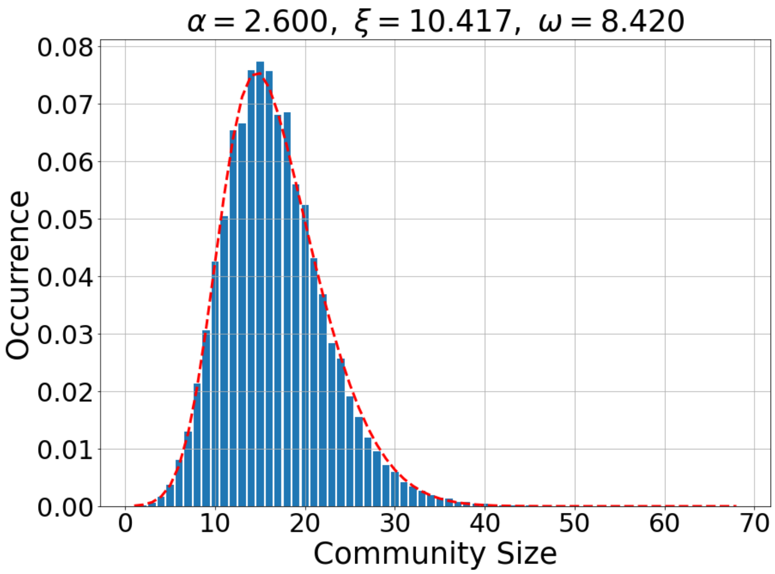

"Then, each community is given a 'manager' node that is placed on a second layer in the graph and attached only to the downstream nodes that are within its assigned community." How is it done? Randomly? How does it look in a real network? Does the "more important manager" (e.g. with a higher degree or some other user importance measure in manager layer) manage bigger groups?

The creation of the manager layer is done via the creation of new nodes, so there is no importance metric for the newly created nodes initially. These new management nodes are spawned as a secondary network, then interconnected to the staff network with interlayer edges only connecting managers to a single downstream community each. The number of nodes in the management layer is exactly equal to the number of partitions created in the staff layer, $m = c = 0.06N$. This is exactly analogous to the interlayer connections used in the empirical datasets, and the intralayer connections between the managers are created independently based on the schemes described (including no connections, full connections, ER, BA, and RGG graphs). For this sort of organizational structure, the size of the downstream group and the degree of the node in the upstream layer are not strongly correlated and forcing such a correlation in the synthetic networks is not indicated by the empirical network structure. Thus, we do not consider any importance measures for managers when assigning communities for them to attach to. The plot here exemplifies this by showing the number of manager connections in the first management layer vs each manager's downstream staff connections in the Line-Org graph. Clarification on the process for building these networks has been added to the paper in lines 170-177, and sample code for reproducing these structures is included in S2 Sample Code.

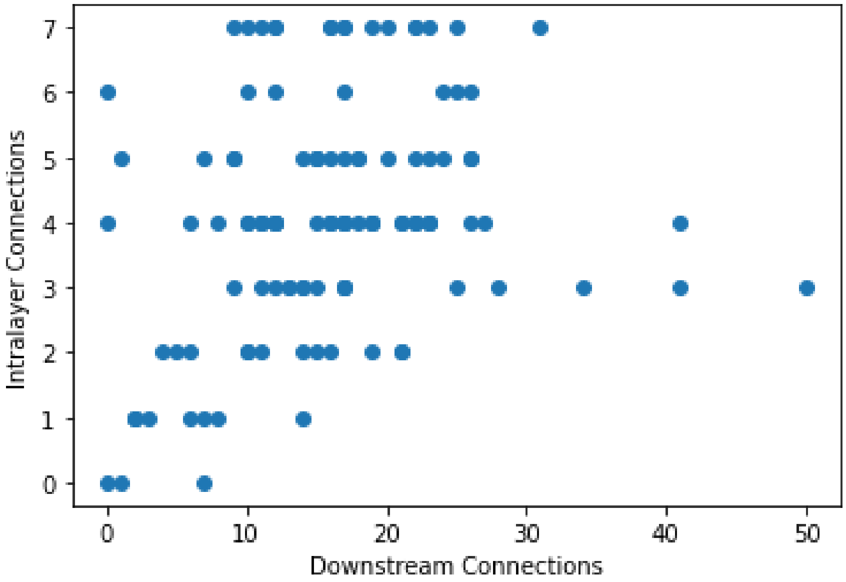

Since some parameters are linked, e.g. removal rate and memory window I would like to know if other combinations of parameters have been tested and what would be the memory window if removal rate would be 0.1 or 0.3? Please include this discussion in the paper.

To maintain the timing symmetry for expected time to removal and memory window used in this paper ($T = 1/r$), removal rates of $r = 0.1$ and $r = 0.3$ would lead to memory windows of $T = 10$ and $T = 3$ respectively (with the latter rounded to the nearest whole number). However, it is important to note that these changes would change the dynamics in favor of longer infection survival times. For the case investigated here with a nonzero removal rate, an absorbing R state ($\rho = 0$ where ρ is the probability of moving from state R to state S) and a memory window of $T > 1$ the system stays within a single class of model as described in (Dodds 2005) for all values of $T > 1$ and $1 > r > 0$. In that paper, they investigate the critical points and phase transitions that arise from different parameter values, including this class of model, and show that with a removal rate of $r = 0.2$, as used in our model, varying the time window simply has the effect of increasing the tail of the simulation and lengthening the time spent in the metastable state. Our value of $T = 5$ is particularly studied there, and is a non-extreme value shown along with $T = 3, 4, 5, 6$ in Fig. 11 in their paper. They show that the effect of the timing window on the persistence time of the infection is a exponential relationship across values of T, where $t_{max} \propto \exp(\alpha T)$, indicating that increasing this timing window simply extends the tail of the simulation. A summary of this explanation has been added to the paper in lines 104-110. Further, in our paper we investigate varying values for the removal rate in this long time limit and show the expected relationship between increased removal rate and decreased long-time survival of the infection without any indication of criticality outside of the extreme cases that would push the system into a different class ($r = 0$, making an SI or SIS model).

Additionally, since many recent papers (e.g. Social Networks through the Prism of Cognition, Complexity, vol. 2021, Article ID 4963903) indicates that people tend to forget, I would like to know if you have considered adding forgetting functions to your model, i.e. that older dosages have a weaker effect on the person?

Regarding the propensity to forget in social modeling, this is one of the key elements and drivers behind the dosage model used here. Having interactions outside of the memory window be lost is a strict form of forgetting, analogous to a stepwise decay function rather than a gradual decay. Further, this model utilizes interactions as the internal clock for individuals

rather than a ground truth global time, allowing for a more heterogeneous forgetting process. As such, the formulation of memory in this model is one way to capture that sort of effect, but could also be extended to a more continuous decay process or tied to connection weights as done in the mentioned paper. A detailed look at possible memory frameworks in modeling is outside the scope of this paper, though a worthy field of study and the power dynamics presented here could contribute non-trivially to the persistence of interactions in individuals. This indication of future work has been added in lines 482-485.

3. **Please add a legend to Fig 2 or add colours to the caption.**

   A legend with colors for different node levels has been added.

4. **Please unify the Y-ax on figures 3a and 3b (scale); 6a with 6b and 6c (formating)**

   The figures have been modified to be more consistent.

5. **Please make all data and code needed for reproduction available, including all synthetic networks generated for simulations which are currently unavailable. You can use CodeOcean or something similar to make your experimental environment capsule.**

   The synthetic networks built here are created using standardized models for random graphs with freely available algorithms for their creation. In particular, in this paper they were built using Python's NetworkX library. The details on the linkages between these standardized layers are described in the text, but to make them more easily reproducible we have updated the segment of the paper that discusses the process of synthetic network creation to be clearer (lines 160-176), and added the supplementary material S2 Synthetic Network Code that can be used to create these synthetic hierarchical structures.

Editor Round One Comments

1. **Are that your manuscript meets PLOS ONE's style requirements, including those for file naming. The PLOS ONE style templates can be found at main body and affiliations.**

   Upon reviewing the style requirements we have made changes to the figure references and file names to properly comply with the journal guidelines.

2. **Thank you for clarifying how the empirical networks data was accessed. Please add the following text to your Methods: Empirical Networks section: 'Access to the data was overseen by the Sandia National Laboratories data governance committee, and all data was fully anonymized before being made available to the authors.**

   The requested text has been added the paper, see lines 163 – 165 on Page 5.

3. **Thank you for stating the following in the Financial Disclosure section:**

   > **This study was funded by Sandia National Laboratories, a multimission laboratory managed and operated by National Technology & Engineering Solutions of Sandia, LLC, a wholly owned subsidiary of Honeywell International Inc., for the U.S. Department of Energy's National Nuclear Security Administration under contract DE-NA0003525.**
   >
   > **The Sandia National Laboratories website is https://www.sandia.gov/ The funders helped procure the data for this work.**

   We note that you received funding from a commercial source:

   > **National Technology & Engineering Solutions of Sandia, LLC**

   Please provide an amended Competing Interests Statement that explicitly states this commercial funder, along with any other relevant declarations relating to employment, consultancy, patents, products in development, marketed products, etc. Within this Competing Interests Statement, please confirm that this does not alter your adherence to all PLOS ONE policies on sharing data and materials by including the following statement: "This does not alter our adherence to PLOS ONE policies on sharing data and materials." (as detailed online in our guide for authors http://journals.plos.org/plosone/s/competing-interests). If there are restrictions on sharing of data and/or materials, please state these. Please note that we cannot proceed with consideration of your article until this information has been declared.
   Please include your amended Competing Interests Statement within your cover letter. We will change the online submission form on your behalf.

   The coverletter has been updated to contain this information and reattached to the new submission. The first round of changes are marked in blue, while the newest additions are in red. In particular, the second to last paragraph now contains the text:

   > This study was funded by Sandia National Laboratories, a multimission laboratory managed and operated by National Technology & Engineering Solutions of Sandia, LLC, a wholly owned subsidiary of Honeywell International Inc., for the U.S. Department of Energy's National Nuclear Security Administration under contract DE-NA0003525. This does not alter our adherence to PLOS ONE policies on sharing data and materials, and the data used in this study is all contained within the supplementary materials of the paper.

Reviewer Round One Comments

1. **It seems that through the paper authors use information, innovation and influence spread as synonyms when, in fact, they are three different sociological phenomena. Thus, please clearly state what is being modeled and evaluate; spread of information or spread of innovation.**

   For this work we focus on the diffusion of innovation, policies, or procedural changes in a corporate environment. The motivating concept is the addition or change of a functional construct that the organization wishes to maximize within its community. In some portions of the paper we had discussed the process as information diffusion, referring to the knowledge of a new technology or policy within the organization, but the spreading dynamics and core modeling effort are built to capture the dynamics of diffusion of innovations, policies, or procedures within the organization. We recognize this verbiage can cause some confusion, and have modified the text of the paper to clarify this and remove insinuations that we are modeling just information (see changes in lines 2, 3, 34, 67, 200, and 416). Additionally, while the paper does not make reference to influence spread directly, we recognize that the increased pressure exerted on social interactions by influential nodes is a primary channel through which innovations diffuse in a hierarchical environment, and one of the major goals of this work is codifying that process.

2. **Having 1, please elaborate on how the selected model (SIR) works in that scenario and how real processes can be translated to mechanisms of the model. For example, if we have spread of information we would have S − do not have information, I − informed, R − forget the information (then maybe it will be better to consider SIS). Innovation case − > S − not adopted, I − adopted, R − forgot/rejected the innovation − > but why one cannot adopt it again? Moreover, if we can reject the innovation why we cannot go straight from I to R based on many neighbouring nodes which already rejected the innovation? Please fully justify the model and the reasoning behind each element of the model.**

   The selection of this model is motivated by the application of organizational innovation spread, with each of the compartments and state transitions being chosen for those purposes. The reviewer's second proposition is exactly right in their understanding of the model state representation. In this work, the S state represents nodes that are unaffiliated with and have not adopted the new innovation or procedure. The I compartment represents adopters that are actively promoting the innovation, and the R compartment represents the rejection of the innovation and its promotion. The R state is made absorbing in the model, meaning that once a node enters that state it cannot leave, to account for the disenchantment and active resistance that is seen in innovation adoption as in (Kahma 2017), as well as to reflect the specific context of management and organizational innovation rejection, where there are many common causes and perspectives to explain lasting innovation rejection as discussed in (Abrahamson 1991).
   The focus of this model is on the social and hierarchical pressures on the adoption of the innovation, and as such the infection has no internal drive for individuals to remain in that state. In application, this means that the modeled innovation would not have a positive efficiency that would allow it to be self-sustaining or resurgent once rejected. Additionally, our time scale and static view of the innovation does not allow for long term effects such as the evolution of the innovation, further indicating the likelihood of long term rejection. Finally, there is no state transition directly from I to R under the assumption of good faith on the part of the agents. A node receiving multiple communications from non-adopters does indeed hasten their path to rejection, and can even prevent their adoption in the first place, but the disenchantment and active resistance that is represented by the R state is reserved for individuals with experience with the innovation. In effect, nodes in state I and nodes in state R behave identically in their outward interactions, both passing on zero dose in their interactions, but only nodes in state R that have negative firsthand experience with the innovation present the active resistance to deny a large social pressure coming either from a superior node or multiple peers. To support

the text of the paper, this explanation has been added at the end of the *Methods: Multi-Layer Dosage Model* section (lines 116 – 136 on page 4) for clarity in the main text of the paper.

Kahma, N., & Matschoss, K. (2017). The rejection of innovations? Rethinking technology diffusion and the non-use of smart energy services in Finland. Energy Research and Social Science, 34, 27–36. https://doi.org/10.1016/j.erss.2017.05.024

Abrahamson, E. (1991). Managerial Fads and Fashions: The Diffusion and Rejection of Innovations. Academy of Management Review, 16(3), 586–612. https://doi.org/10.5465/amr.1991.4279484

3. **Having 1 and 2, please clearly justify selected parameters for example, why a network is clustered into 60 clusters (is it always 60 or depends on the network size?, why 60?)? Why the probability of transition to R is 0.2, why not 0.1 or 0.5, what will be the effect on the process depending on selected probability? Why 370hours of collaboration and not 100 or 500? etc.**

There are two different types of model parameters used in the paper: those that control the behavior of the dosage model spreading mechanism, and those that control the structure of the synthetic networks used. For the synthetic network structure, choices are uniformly made to be consistent with the empirical networks built and used for this work to ease comparison between the structures. For instance, the base layer of the synthetic networks is of size $N = 1000$ nodes and divided into 60 communities that are then assigned 'manager' or level 2 nodes. The number 60 is derived from 6% of the population, which is the size of the first management layer in the empirical networks relative to the staff layer (115 nodes and 1787 nodes respectively). The number of communities is fixed across runs, the Fluid Communities algorithm used is stable in its ability to partition to an exact number of communities for reproducibility, important in this case as adding extra managers into some runs but not other obviously proffers an undue advantage. The description of the synthetic networks in *Methods: Synthetic Networks* (lines 150-154 on page 4) has been updated to clarify this, and this method is also continued in the new exploration of system sizes to modulate the number of managers with the size of the system. Further, we develop the synthetic networks to hold an average degree $\langle k \rangle = 20$ to mirror that found in the empirical networks. Similarly, the threshold of 370 coworking hours in the Project graph is chosen in order to present an identical overall average degree as the Line-Org graph ($\langle k \rangle = 20$) as mentioned in the section *Methods: Empirical Networks: Project Graph.* Since the Line-Org network is a naturally more rigid and well defined construct, and a limiting threshold had to put upon the Project network in order to support an unweighted graph, we chose to utilize the structure of the Line-Org network as our limiting edge factor the Project graph to improve comparability.

Separately, parameters chosen for the dynamics of the dosage model are done so in order to as closely match the motivating application as possible while providing a tractable and reasonable simulation for our structural analysis. We do not propose a full parameter sweep or generalization on this model, as that was discussed extensively by the original authors of the model in their paper (Dodds 2005).

However, the parameters are chosen to be reasonable and useful within context, and many can be seen to simply have the effect of lengthening or shortening the timescale of the simulation when taken in conjunction. For instance, we utilize a removal rate of 0.2 in our model in conjunction with our memory window of $T = 5$ timesteps. This is done such that when a node's total dosage drops below the threshold, they have, on average, 5 timesteps to receive a new dose before abandoning the innovation, providing a level of symmetry to the infection and removal process. Further, we test sensitivity on the removal rate due to its criticality to the simulation, shown in Fig. 6C. This analysis shows the sensitivity of the value beginning to slow at the chosen value of $r = 0.2$, although obviously increasing the removal rate has the expected effect of lowering the overall spread and long-term viability of the infection. These clarifying

points on the selection of various values have been added to the *Methods: Multi-Layer Dosage Model* section (lines 116 – 136 on page 4).

Dodds, P. S., & Watts, D. J. (2005). A generalized model of social and biological contagion. Journal of Theoretical Biology, 232(4), 587–604. https://doi.org/10.1016/J.JTBI.2004.09.006

4. **Selected networks are all of the same size 1000-1800 nodes which do not allow for results generalization. Please use both smaller and bigger networks to address both SMEs and large corporations at least for simulated data. For real networks, please use other "company/institution" datasets like Manufacturing emails (Radoslaw Michalski, Sebastian Palus, and Przemyslaw Kazienko. Matching organizational structure and social network extracted from email communication. In Lecture Notes in Business Information Processing, volume 87, pages 197–206. Springer Berlin Heidelberg, 2011.), Enron emails and Enron structure (Palus, S., Brodka, P., & Kazienko, P. (2011). Evaluation of organization structure based on email interactions. International Journal of Knowledge Society Research (IJKSR), 2(1), 1-13.) and others.**

   Generalizing the results to multiple system sizes is a very important consideration which we had neglected with our focus on the empirical dataset we had built. To remedy this, we have added a new figure, Fig. 3(B), containing the results of running our model on the synthetic version of our network with various system sizes ranging in size from 200 nodes to 8000 nodes. In this analysis we show that the results are stable across system sizes in the synthetic network. Unfortunately, due to the difficulty of obtaining similar suitable datasets to our empirical network, we are not able to do such a comprehensive sweep on real-world data sizes. This sort of ground truth hierarchical data is difficult to find in the public sphere, and one of the goals of our publication is the production and release of new hierarchical network data that is an order of magnitude larger than those currently available to support future work. However, the polish manufacturing data that the reviewer points out is well suited to this work, and we have included analysis of that network in *S1 Appendix*, showing that our results hold when the model is applied to this new dataset. Unfortunately, we are not able to repeat this analysis using the suggested Enron dataset due to its lack of a ground truth reporting structure and hierarchy. The dataset contains only vague fields for position within the company, and no reporting lines to build a true organizational map, a crucial element for our hierarchical spreading process. Since this dataset presents little in the way of ground-truth structure, and its size is extremely similar to the Polish manufacturing data, we chose to focus our efforts on results reproduction in the Polish manufacturing network, which can be found in *S1 Appendix*.

5. **Selected approach for seed selection does not allow for (1) easy comparison of results between networks of various sizes and (2) results reproducibility. For (1), please consider using a percentage of a network as seeds (e.g. 1% or 0.5% of nodes will be seeds) instead of a fixed number of seeds. For (2), please consider using some seed selection strategy instead of random selection, e.g. top % of nodes with the highest degree. There is many seed selection strategies for multilayer and single layer networks (Erlandsson, F., Bródka, P., & Borg, A. (2017, November). Seed selection for information cascade in multilayer networks. In International Conference on Complex Networks and their Applications (pp. 426-436). Springer, Cham.). The most useful in case of the problem presented in the paper and high cauterization of nodes might be community structure based method like (He, J.-L., Fu, Y. & Chen, D.-B. A novel top-k strategy for influence maximization in complex networks with community structure. PloS one 10, e0145283 (2015).). Please also use simple degree as a benchmark. Please consider using some early adopters mechanism to introduce the real appearance of innovation in the company, which is usually done by people with some key characteristic (leaders, influencers, innovators) rather than random people.**

We have addressed the problem of seed sizes being reported as absolute numbers, replacing that with percentages of the network throughout the paper and normalizing across experiments of a minimal seed and one of 1.5% the network size. Especially in light of the increased focus on various network sizes, this is crucial for accurate comparisons between simulations. The chosen values now utilize (1) a minimal seed, where regardless of network size we simply seed the smallest possible fraction (a single node), and (2) a seed size of 1.5% of the network. This value is chosen as it is the largest fraction of the network that allows us to still seed into the third level (management level 2) of the empirical graphs. Since that level is 1.5% of the empirical networks, it represents a good maximal seed for investigations across levels.

The use of non-random seeding is an important point to understand optimal spreading practices, and as such we have added a new section *Results and Discussion: Empirical Networks: Seed Selection Strategies* (lines $365-411$ on pages $11-13$). In this section we repeat the analysis of the maximum adoption point for three different seed selection strategies: simple degree, VoteRank, and KCore. These three methods are chosen as leaders in classical seed selection for spreading processes, and are used as a comparison to our random selection strategies. Fruther, we recognize the importance of the paper by J.-L. He mentioned in the review, and have included reference to it in our paper since it has high overlap with our goal. The method they propose is meant to assume hierarchy in community based systems and identify leaders within groups to seed into, a method that partially reconstructs the information that is explicitly provided in our data via the management scheme. Indeed, if considering the empirical networks we study here, it can be intuitively understood that moving up a seed level in our networks provides an explicitly labeled leader of the community below. In the Line-Org network this must be the case, as each lower node is fully connected within their department and to the manager above. Therefore, if we select that community to find a leader, and utilize a centrality measure to determine the leader, the manager must be the 'leader' of that group, as they are connected to all the downstream nodes as well as to other manager nodes. They have both higher degree and are more central in the network, and since no manager leads more than one group, there is no issue with normal community overlap. The same is true, albeit less explicitly, in the P/T network, where managers are shown to have much higher centrality and degree than staff nodes, leaving them highly connected within communities and more central than other nodes within the community in the overall network. This discussion is included in the paper lines $405-413$ on pages $12-13$.

6. **What was the dosage threshold for each node? Was it always the same or it was selected randomly in each run. If randomly each time how you compare results between two networks if we have random seeds, random thresholds and random transition to R each time? Please consider coordinated execution approach similar to one used in (Jankowski, J., Szymanski, B. K., Kazienko, P., Michalski, R., & Bródka, P. (2018). Probing limits of information spread with sequential seeding. Scientific reports, 8(1), 1-9.) where you generate for example 10 000 instances of nodes threshold and R transitions plus add the seeds selected according to some heuristic/strategy. Then we can compare the results between networks for each instance and see what affect the network topology and/or seed selection strategy have on the spreading process.**

   In the original paper, all dosages and thresholds were randomly assigned at runtime based on a gaussian distribution for each. We utilized Monte Carlo sampling over many runs to achieve reproduceable results, a method acknowledged in the mentioned paper by Jankowski. However, in recognition of the high number of random variables and request to investigate specific seeding algorithms, it is natural to utilize a more consistent approach for ease of comparison. To this end, the new analysis of various seeding algorithms is performed with controlled random seeds such that each simulation is run on an identically initialized network and utilizes identical transmission pathways and doses. The resultant analysis is contained in the new

section *Results and Discussion: Empirical Networks: Seed Selection Strategies* (lines $365-411$ on pages $11-13$).

7. **Please evaluate if results are statistically significant.**
   Monte Carlo simulations such as those run here have no inherent simulation bias, since the expectation value of any individual run is the (unknown) ground truth average. The standard error of the estimator, however, is equal to the ground truth error times the inverse root of the sample size. Thus, minimizing the standard error is done by having a sufficiently large sample size, leading to stabilization of the means observed. We include this analysis here, where we show that the averages have long since stabilized by the time 1000 runs are completed, that there is no drift when extended to 2000 runs, and that the variance between batches is very small compared to the scale of the network and hierarchy effects.

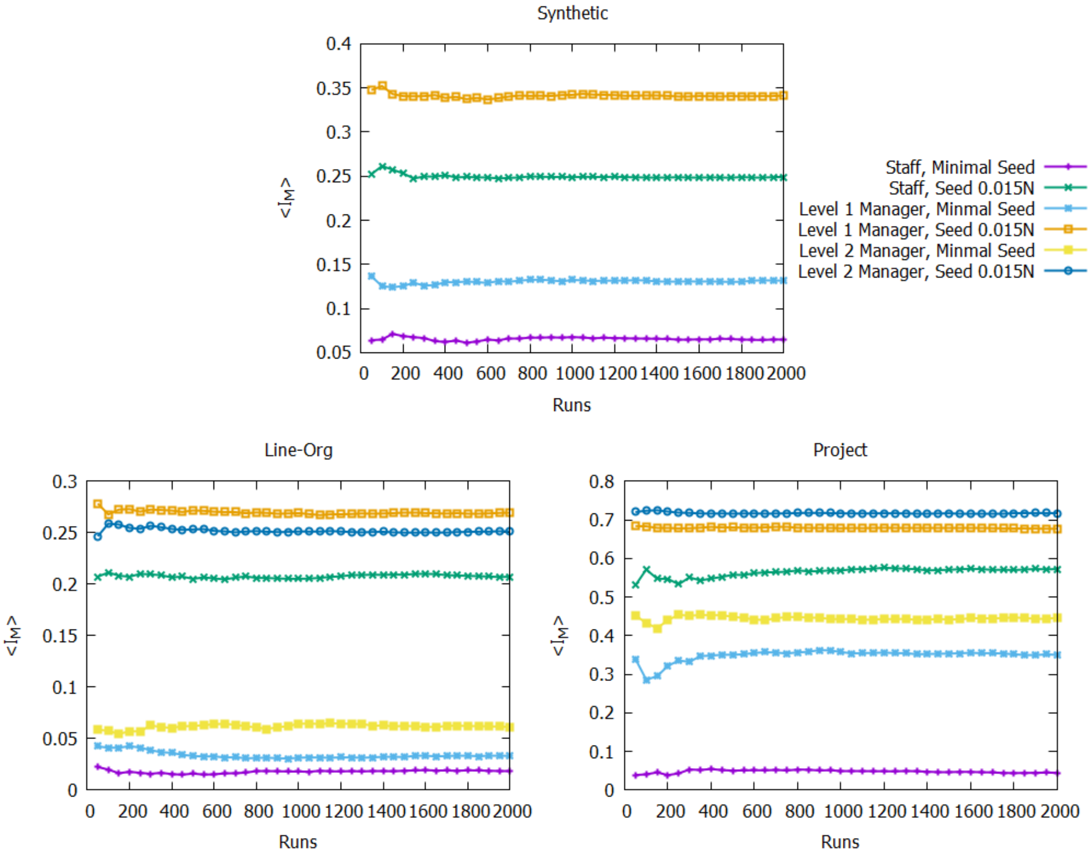

8. **What is the difference between Dosage SI model and Linear threshold model –on the first glance, it looks the same. If they are the same, please consider noting that in the manuscript.**

   The two models are very similar, but the Dosage SI model is a pairwise interaction model while the Linear Threshold model has nodes broadcasting to all neighbors every timestep utilizing edge weights to determine total dosage. The Dosage SI model does not utilize edge weights and is done on unweighted networks such as those contained in this paper. Each interaction is considered a micro-timestep where a speaker-listener pair is selected from the eligible pool of nodes. A full system timestep consists of N micro-timesteps, without guarantee that each node will activate during a single system timestep. This framework allows not just for the lack of edge weights, but also allows nodes to proffer zero doses, effectively dampening the spread by taking up space in other node's memory. In this way nodes in compartment R remain active within the system by actively opposing the innovation, and the added memory allows for a more accurate social contagion framework for innovation spread.

9. **Please unify the naming convention in the paper, e.g. sometimes it is multilayered, sometimes multi-layered.**
   We have gone through the paper and fixed these inconsistencies, thank you.
